# DNA Modification Patterns within the Transposable Elements of the Fig (*Ficus carica* L.) Genome

**DOI:** 10.3390/plants10030451

**Published:** 2021-02-27

**Authors:** Gabriele Usai, Alberto Vangelisti, Samuel Simoni, Tommaso Giordani, Lucia Natali, Andrea Cavallini, Flavia Mascagni

**Affiliations:** Department of Agriculture, Food and Environment, University of Pisa, 56124 Pisa, Italy; gabriele.usai@agr.unipi.it (G.U.); alberto.vangelisti@agr.unipi.it (A.V.); samuel.simoni@phd.unipi.it (S.S.); tommaso.giordani@unipi.it (T.G.); lucia.natali@unipi.it (L.N.)

**Keywords:** *Ficus carica* L., LTR-retrotransposon, DNA-transposon, N6-methyladenine, N4-methylcytosine

## Abstract

Transposable element activity can be harmful to the host’s genome integrity, but it can also provide selective advantages. One strategy to cope with transposons is epigenetic control through DNA base modifications. We report the non-canonic DNA modification dynamics of fig (*Ficus carica* L.) by exploiting high-quality genome reference and related N4-methylcytosine (4mC) and N6-methyladenine (6mA) data. Overall, 1.49% of transposon nucleotides showed either 4mC or 6mA modifications: the 4mC/6mA ratio was similar in Class I and Class II transposons, with a prevalence of 4mC, which is comparable to coding genes. Different percentages of 4mC or 6mA were observed among LTR-retrotransposon lineages and sub-lineages. Furthermore, both the *Copia* and *Gypsy* retroelements showed higher modification rates in the LTR and coding regions compared with their neighbour regions. Finally, the unconventional methylation of retrotransposons is unrelated to the number of close genes, suggesting that the 4mC and 6mA frequency in LTR-retrotransposons should not be related to transcriptional repression in the adjacency of the element. In conclusion, this study highlighted unconventional DNA modification patterns in fig transposable elements. Further investigations will focus on functional implications, in regards to how modified retroelements affect the expression of neighbouring genes, and whether these epigenetic markers can spread from repeats to genes, shaping the plant phenotype.

## 1. Introduction

Transposable elements (TEs) are mobile sequences generally accounting for the largest fraction of the genome’s repetitive component, i.e., the amount of DNA with no obvious functional–regulatory or protein-coding relevance for the organism. TEs are divided into two classes in eukaryotes: Class I and Class II. Class I TEs (retrotransposons, REs) use a ‘copy-and-paste’ strategy to transpose, which implies the generation of an RNA intermediate to be retrotranscribed and inserted in the genome; Class II TEs (DNA transposons) move through DNA excision with a ‘cut-and-paste’ mechanism [1]. Both types are sub-divided into orders and lineages, based on sequence homology and on whether or not the TEs encode their own transposition machinery. The most abundant RE order in plant genomes are long terminal repeats REs (LTR-REs), which are made of the sequences encoding the transposition machinery, including a GAG domain, which is committed to the production of virus-like particles, and a polyprotein involved in the retrotranscription and insertion of the DNA into the genome, flanked by two long terminal sequences, which are identical at the time of insertion. These kinds of REs make up the main portion of many genomes [2,3], and are subdivided into two major superfamilies, called *Gypsy* and *Copia*, that differ in the position of the protein domains within their encoded polyprotein [1]. In turn, the superfamilies can be classified into several major evolutionary lineages [4,5]: eight for *Copia,* and three main lineages for *Gypsy* [6,7,8]. Although, in recent years, it emerged that TEs can provide selective advantages to the host, at the same time, their mutagenic properties represent a risk for the integrity of the host genome [9,10,11]. In response, genomes have evolved several layers of defense to contain TE activity.

One of the strategies implemented by host genomes to counteract the proliferation of repeated elements, associated with chemical modifications of DNA and/or histone [12], is methylation: the epigenetic control of loci is in fact often linked to the silencing of TEs as part of a genome defense system [13]. TEs are predominantly located in the heterochromatin regions of the chromosomes, such as the centromeres [14]. A high level of cytosine methylation is commonly detected in the repetitive DNA [15], especially as of 5-methyl cytosine (5mC) [14]. It has been reported that, in Angiosperm evolution, when the genome size increases, largely due to the proliferation of LTR-REs, the overall level of DNA methylation increases [16]. Transposon-related DNA modifications can also affect the host gene expression, chromosome conformation, and the differentiation of pericentromeric heterochromatin and distal euchromatin. One of the simplest consequence of TE silencing is the effect on the transcription of the host when the silenced TE is located within or nearby a gene: in the oil palm, the methylation level of a TE located within a key gene involved in the flowering process affects the whole’s fruit oil production [17].

After their insertion, TEs are strongly targeted by DNA methylation, resulting in changes in the flanking chromatin regions, as demonstrated in several plant species [18,19,20,21]. The randomness of TE transposition and the subsequent methylation that may be triggered can lead to the origin of epialleles and other major effects, which are heritable over generations [22,23].

Besides the addition of a methyl group to the carbon-5 position of cytosine to produce a 5mC, other DNA base methylation can occur in the genomes. Recent advancements of sequencing technologies—such as the single-molecule real-time (SMRT) sequencing developed by Pacific Biosciences (PacBio)—are making it possible to discover unconventional epigenetic modifications through the genomes, including N4-methylcytosine (4mC) and N6-methyladenine (6mA) modifications [24]. Unlike next-generation sequencing (NGS) technologies, PacBio sequencing also allows us to detect native epigenetic modifications thanks to its innovative approach, by monitoring the time between the base incorporations in the read strand. The presence of methylic groups on the cytosine N4 and adenine N6 is associated with distinct time patterns, allowing us to call these types of modified bases [24].

These sequencing approaches have recently allowed the publication of specific studies on plant species highlighting the patterns and structural distribution of 4mC modifications [25,26], which—to date—has mainly been studied in bacterial genomes [27,28,29]. These and other findings may lead to new future discoveries on the effect of 4mC modifications on the tuning of both gene and TE expression. Furthermore, 6mA modifications in eukaryotes have recently received much more attention than in the past, and this type of modification has been found to be a potential epigenetic marker in several plant genomes [30,31]. Nevertheless, because of the limitations of current technologies for 6mA detection and the sophistication of the 6mA regulatory mechanism, the specific distribution of the motifs and the potential effects of such modifications in plant genomes remain poorly understood.

Fig (*Ficus carica* L.), one of the oldest known domesticated species, has a relatively small genome (~356 Mbp) that was recently sequenced and assembled at a high-quality level by using the SMRT sequencing technology [26]. The genome sequence revealed that the repetitive fraction amounted to 37.39%; REs accounted for 75.69% and DNA transposons for 15.05% of the repetitive DNA [26]. The relatively small genome size and the abundance of reliable genomic resources make the fig tree a new model species for fruit trees.

Here, we present a study that—by exploiting the available fig genomic data on 6mA and 4mC—addresses many facets related to the dynamics of the DNA modifications of the fig repetitive component. The focus of our study is the characterization of the unconventional methylation patterns associated with repeats, which are known to be more subject to epigenetic control via 5mC modifications, in order to probe the extent to which unconventional DNA modifications epitomize the conventional ones, and whether functional similarities between the two can be hypothesized.

This structural analysis includes: (a) measuring the level of DNA modifications related to the fig’s transposable elements; (b) exploring DNA modifications in relation to the structure of full-length LTR-REs; (c) measuring the frequency at which the most methylated TEs are located near the centromeres.

## 2. Materials and Methods

### 2.1. Sequence Collection

Fig genome-wide TE distribution and the overall base modification landscape were intersected to perform a structural analysis, in order to highlight the distribution and putative correlations among these data and the proximal protein-coding genes. In particular, we focused on 4mC and 6mA modifications and their distribution patterns. The analysis was performed using data previously published by Usai et al. [26]. The fig genome assembly (project number PRJNA565858) was downloaded from the NCBI site (https://www.ncbi.nlm.nih.gov/; 1 September 2020), while the TEs, protein-coding genes, and genome-wide 4mC and 6mA modification datasets were downloaded from Usai et al. [26]. As 4mC and 6mA produce strong kinetic signatures, the manufacturer’s protocol recommends a coverage of 25x per strand [24]. The 4mC and 6mA detection on fig was performed using a coverage of about 74x [26]; therefore, the process can be considered confident. Furthermore, the average coverage of the mapped PacBio sequences was calculated for each chromosome using the genomeCoverageBed function of BEDtools v2.27.0 [32].

### 2.2. Genome-Wide Transposable Elements Distribution Analysis

The annotated repeat library of fig TEs was used to mask the whole fig assembly by using RepeatMasker v4.0.3, with the default parameters [33]. The coordinates of the masked regions were intersected with those of the genome-wide 4mC and 6mA modification datasets using BEDtools, allowing us to calculate the number of modified bases on the total masked regions associated with the various TE superfamilies and lineages. Similarly, the overall representation of the associated motifs in these regions was also investigated.

### 2.3. Full-Length LTR-REs Modification Analysis

The 4mC and 6mA modification levels of the annotated full-length LTR-REs of the *Gypsy* and *Copia* elements were investigated. The differences among the 4mC and 6mA modification level averages of the LTR-RE lineages were tested using ANOVA, with Tukey’s method for the post-hoc analyses (R package ‘agricolae’ [34]). A separate test was performed, keeping separate the *Gypsy* and *Copia* superfamilies.

In order to study the distribution of 4mC and 6mA along the full-length LTR-REs, the elements were individually split into five bins according to the coordinates of their genome localization, corresponding to a 1-kbp window upstream of the 5’-LTR region, to the 5’-LTR, to the GAG-POL region, to the 3’-LTR region, and to a 1-kbp window downstream of the 3’-LTR region. The coordinates of the five bins were intersected with those of the genome-wide 4mC and 6mA modification datasets using BEDtools. The data were normalized based on the length of the elements. The statistics were conducted using ANOVA, with Tukey’s method for the post-hoc analyses.

### 2.4. Distribution Analysis of Methylated LTR-REs According to Their Centromere Proximity

The centromeric regions were identified by masking the 13 fig pseudo-chromosomes with the centromeric tandem repeat identified by Usai et al. [26]. The masking process was performed using RepeatMasker, with the default parameters. The central coordinates were used as starting points from which the 3 Mbp forward and reverse intervals were considered up to the end of the pseudo-chromosomes. The 3 Mbp intervals were intersected with the full-length LTR-Res, and the number of the modified bases was calculated. The binning and the intersection processes were performed using the window option of BEDtools.

### 2.5. Analysis of the Proximity of Methylated LTR-REs to Genes

In order to estimate the number of genes in proximity to each full-length LTR-RE, the coordinates of the *Gypsy* and *Copia* elements were compared to those of the known protein-coding genes of the fig genome using the window option of BEDtools, considering a 50-kbp symmetric window upstream and downstream of each element. TEs may be associated with the regulatory elements of genes, and can therefore influence gene expression. It is known that the effect of the presence of a TE on gene expression may occur at variable distances [18,19,20,21]. There is a notable case of a TE inserted tens of thousands of base pairs upstream of the *teosinte branched1* (*tb1*) gene of maize, which acts as an enhancer of gene expression [35]. Accordingly, the 50-kbp window was selected as a reasonable range to investigate putative TE-affected genes.

## 3. Results

### 3.1. Genome-Wide DNA Modifications of the Fig Repetitive Component

Exploiting the existing 6mA and 4mC genomic data of *F. carica* obtained from Usai et al. [26], we first focused on the evaluation of the genome-wide unconventional base modification landscape and its distribution patterns in the fig DNA-TEs and REs. The 74x coverage data used by Usai et al. [26] assured us a confident 4mC and 6mA detection, confirmed by an average coverage of the mapped sequences >55% for each chromosome.

**Figure 1:**
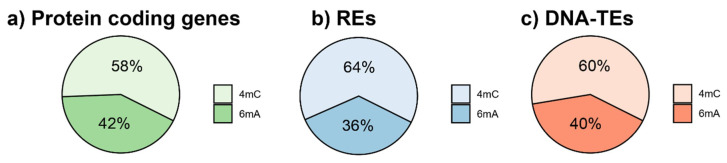
Distribution of the modified bases (4mC and 6mA) in protein coding genes (**a**), REs (**b**), and DNA-TEs (**c**).

Overall, 1.49% of the nucleotides in the fig interspersed repeats displayed a 4mC or 6mA DNA modification [26]. The ratio between the 4mC and 6mA frequency was very similar in the protein coding genes, Class I and Class II TEs, with a prevalence of 4mC over 6mA (Figure 1).

Similar frequencies of 4mC and 6mA were observed in different TE classes, orders, and lineages, with 4mC being more represented in all of the analysed TE types, despite the lower GC content (Table 1). The only exceptions were the SINE REs, in which the 6mA were slightly more represented than 4mC. The same trend was also observed at the gene level, which showed a 4mC/6mA ratio of 1.39, and a slightly higher GC content (42.54%).

The presence of the most significantly enriched 4mC motifs (CG, CHG and CHH; where H is adenine, cytosine or thymine) and 6mA motifs (ANHGA, GAGG, CAAG ACCT and A; where H is adenine, cytosine, or thymine, and N can be any nucleotide), identified by Usai et al. [26] in the fig genome, were tested for both Class I and II TEs (Supplementary Table S1).

### 3.2. 4mC and 6mA DNA Modifications in Fig Full-Length LTR-REs

In order to study the levels of unconventional DNA modifications in LTR-REs, we focused on full-length elements. We measured the percentage of base modifications on a library of 1786 full-length elements belonging to the *Gypsy* and *Copia* LTR-RE superfamilies, showing a significant difference in relation to the superfamily and to the lineage of the element. The *Gypsy* full-length LTR-REs showed a mean percentage of 4mC and 6mA higher than the *Copia* elements (2.83 +/− 0.05% vs. 1.84 +/− 0.04%, respectively).

**Figure 2:**
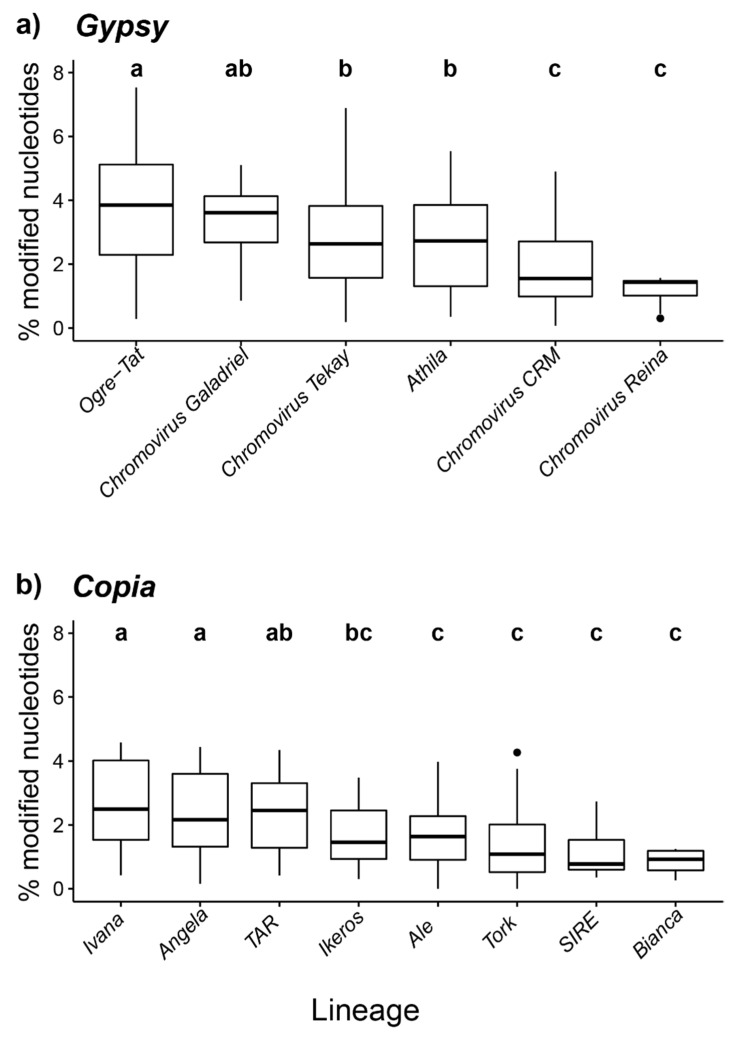
Percentage of modified nucleotides (4mC and 6mA) in fig full-length LTR-REs. The distributions of the percentage of the modified nucleotides are reported across different lineages of full-length *Gypsy* (**a**) and *Copia* (**b**) LTR-REs. The letters above each box-plot indicate the significance ranking (*p <* 0.05) according to Tukey’s test; the dots represent outlier values.

For the *Gypsy*-related LTR-REs, we analysed the percentage of modified bases in 1165 REs belonging to three main lineages—*Chromovirus*, *Ogre-Tat*, and *Athila* [7]—revealing a significant effect of lineages (*p* < e^−16^); the *Chromovirus* lineage, being the most abundant, was investigated at the sub-lineage level. The *Ogre-Tat* elements showed the highest 4mC and 6mA proportions with *Chromovirus/Galadriel* LTR-REs, while the lowest unconventionally methylated elements belonged to the *Chromovirus/Reina* sub-lineage (Figure 2a); however, there were not significant differences between the *Athila* lineage and the *Chromovirus/CRM* sub-lineage.

The 621 full length LTR-REs of the *Copia* superfamily were analysed, relating the modified bases in the 8 lineages: *Ale*, *Angela*, *Bianca*, *Ikeros*, *Ivana*, *SIRE*, *TAR* and *Tork* [7]. Overall, the lineages significantly explained the variance of the modified bases (*p* < e^−14^). The post-hoc analyses revealed the ways in which the elements belonging to the *Ivana* lineage showed the highest level of 4mC and 6mA modifications, together with the *Angela* and *TAR* LTR-REs. The lowest value was assigned to the *Bianca* LTR-REs, which showed similar levels of such DNA modifications with the *Ikeros-*, *Ale-*, *Tork-* and *SIRE-*related elements (Figure 2b).

### 3.3. Unconventional DNA Modifications in Fig Full-Length LTR-Res, and Their Proximity to Centromeric Regions

In order to understand whether or not the DNA modification (4mC and 6mA) levels are differentially distributed within the LTR-REs, we generated the average base modification patterns along all of the full-length LTR-RE of the *Gypsy* and *Copia* superfamilies in the reference sequence and, additionally, along the up- and downstream 1-kb regions.

**Figure 3:**
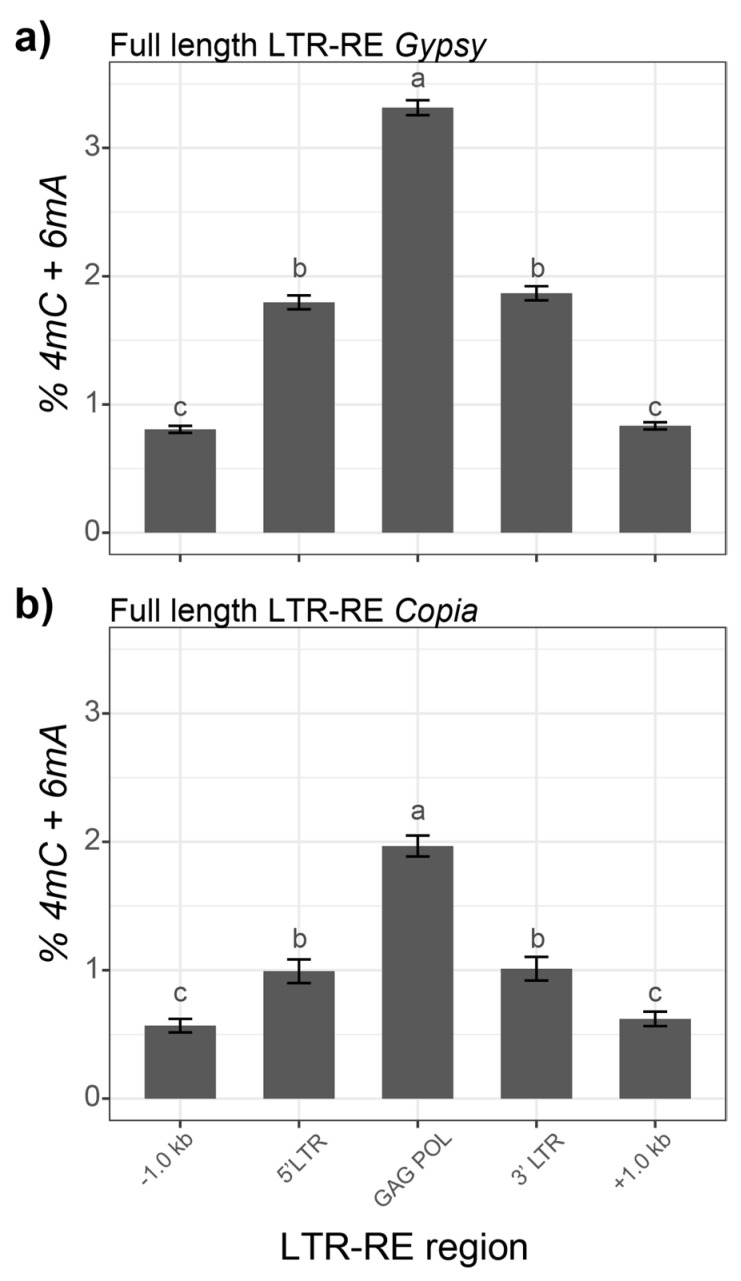
Percentages of 4mC and 6mA in full length *Gypsy* LTR-REs (**a**) and *Copia* LTR-REs (**b**), including a 1-kbp window upstream and downstream of the LTR sequences. The bars report, for each bin, the average of the modified nucleotide in the corresponding regions of the REs, with the standard error included as the error bar. The letters above each bar indicate the groups assigned by Tukey’s test (*p <* 0.05).

We then computed the proportion of modified nucleotides (4mC and 6mA), keeping separate the LTR region and the coding region of the elements. The results of this analysis indicated how, for both *Copia* and *Gypsy*, higher modification rates were measured in the LTR and coding regions, with lower amounts in the 1-kbp regions upstream and downstream of the REs. The analysis of variance indicated a significant effect (*p* < e^−16^) of the RE region on the methylation level, for both superfamilies. A post-hoc investigation using Tukey’s test revealed that, for both superfamilies, there was higher methylation in the GAG/POL related regions than in the 5′ and 3′ LTR, and that the RE upstream and downstream regions were less methylated than the RE (Figure 3a,b).

The distribution of full-length LTR-REs was evaluated in proximity to the centromeric regions of fig pseudo-chromosomes [26], in intervals of 3 Mbp, starting from the centromere and proceeding upstream or downstream.

**Figure 4:**
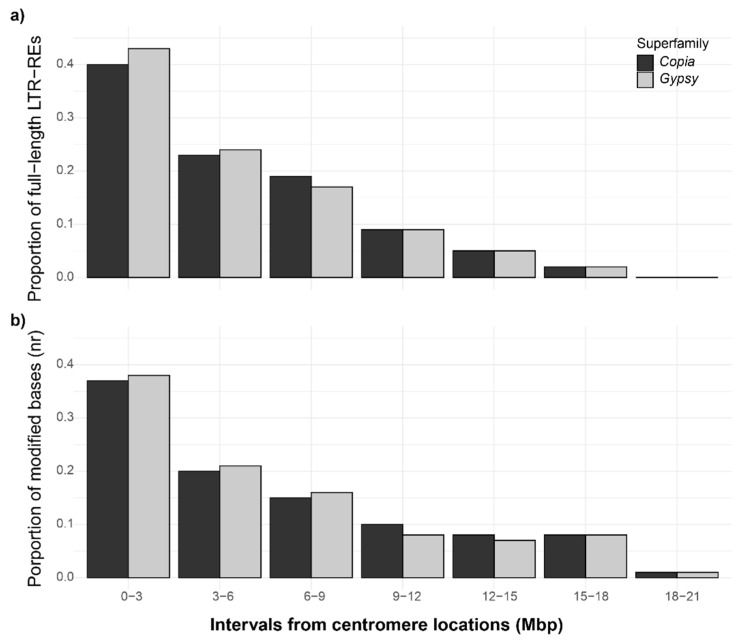
Bar plots of the full-length LTR-RE (**a**) and modified bases (4mC and 6mA, b) distributions according to their proximity to the centromere of the 13 *F. carica* pseudo-chromosomes in the *Copia* and *Gypsy* superfamilies. Starting from the center of the centromeric regions (point 0–3 on the x axis), 3 Mbp bins upstream, downstream, and reverse were considered. The distributions of each superfamily were normalized based on the total number of elements (**a**) or the total number of modified bases (**b**).

As expected, the density of the LTR-REs was higher near the centromeric regions (Figure 4a). We also observed a high abundance of *Chromovirus* elements in the centromeric regions which are typically abundant in these structures (data not shown) [7].

The profiles of the mean number of the modified bases associated with the full length LTR-REs in the aforementioned intervals are reported in Figure 4b. On average, the absolute number of LTR-RE modified bases ranged between ~10,800 and 400; after normalizing each superfamily for the total number of bases, no differences were observed in this respect between the *Gypsy* and *Copia* elements (Figure 4b).

### 3.4. Analysis of the Gene Proximity to Methylated LTR-REs

**Figure 5:**
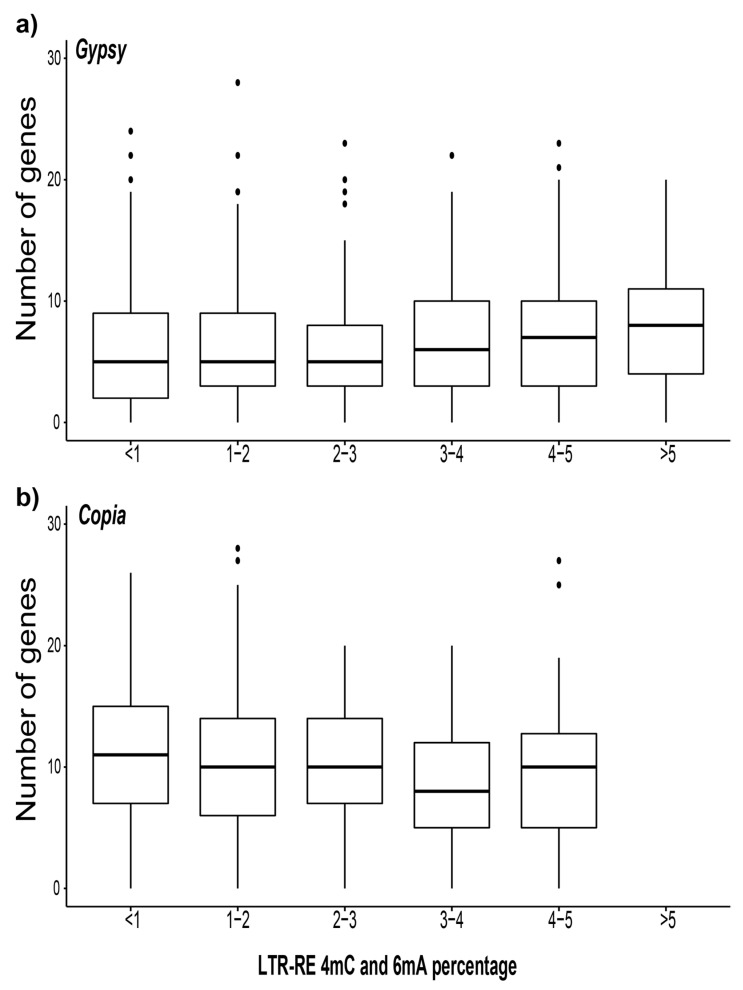
Number of genes proximal to the *Gypsy* (**a**) and *Copia* (**b**) full-length LTR-REs, subdivided into classes according to their unconventional methylation percentage. The LTR-REs were clustered in six groups according to their methylation levels (from <1% to high, >5%); the dots represent outlier values.

In order to test whether or not LTR-REs exhibited different unconventional methylation levels when their location falls close to coding genes, the N4-cytosine plus N6-adenine methylation level was computed by measuring the percentage of 4mC and 6mA inside each full-length element, and then counting the number of proximal genes, which were defined as genes within 50,000 bp of the LTR-RE. The LTR-REs belonging to the *Gypsy* and *Copia* superfamilies were classified into six groups according to their N4-cytosine and N6-adenine methylation levels, ranging from low (<1%) to high (>5%) (Figure 5a,b).

Of note, the unconventional methylation levels of the *Copia* elements did not exceed 5%. The median number of proximal genes for *Copia* LTR-REs was approximately 10, regardless of the methylation level. The number of genes proximal to *Copia* elements did not change among the LTR-RE groups with different methylation levels. On the other hand, the *Gypsy* elements showed a lower number of proximal genes. Although the variation was not significant, the number of genes proximal to *Gypsy* elements tended to increase with the N4-cytosine and N6-adenine methylation level of the elements. The unconventional base modification levels of genes lying close to highly-methylated LTR-REs (i.e., those with a proportion of modified nucleotides higher than 4%) were measured. A total of 2326 genes were identified, i.e., 2000 lying close to highly-methylated *Gypsy* elements, and 326 lying close to the highly-methylated *Copia* elements. Among them, only nine genes were in turn highly methylated (i.e., genes with a proportion of modified nucleotides higher than 4%), seven of which were close to *Gypsy,* and two of which were close to *Copia* elements, respectively. The complete list of the genes lying close to the highly-methylated LTR-REs is reported in Supplementary Table S2.

## 4. Discussion

In the last few years, the emergence of third-generation sequencing technologies—including single-molecule real-time (SMRT) sequencing—allowed us to achieve not only genome sequences with higher levels of completeness but also to identify epigenetic DNA modifications, including the presence of unconventionally-methylated bases, such as N4-methylcytosine (4mC) and N6-methyladenine (6mA) [24].

*Ficus carica* has a relatively small genome (356 Mbp, [36]), of which transposable elements represent roughly one third [26]. The high-quality genome assembly of fig obtained with SMRT [26] made available genomic data such as the sequences of its repeatome, including a collection of full-length LTR-REs, and the characterization of the N4-cytosine and N6-adenine methylation profiles along the reference genome. In a previous work by Usai et al. [26], the overall genome-wide frequency of 4mC and 6mA modifications was described, comparing the patterns of such modification in genes and repeat regions, but without a specific focus on the latter. Exploiting these genomic resources, this work provides the first insight into the dynamics of the DNA modifications of the repetitive component in the genome of a higher plant.

Classical 5-methyl cytosine (5mC) DNA modification has often been related to the repeatome of eukaryotic genomes [15]. In this study, we focused on the occurrence of 4mC and 6mA. In the fig genome, 1.49% of the DNA bases of the repeatome showed either 4mC or 6mA modifications [26], with an overall prevalence of 4mC over 6mA (Figure 1, Table 1). The importance of these two types of DNA modifications have been magnified in studies of bacterial genomes: in fact, 6mA and 4mC play a role in the balance of restriction-modification systems [27]. In eukaryotes, recent evidence highlighted 6mA as a potential epigenetic marker [31,37,38,39], possibly affecting the expression of genes [40].

To the best of our knowledge, there is a scarcity of studies regarding the different methylation patterns that can be examined in REs belonging to different superfamilies and/or lineage. In fact, while the important contribution of repetitive DNA for the genetic variation of plants is acknowledged [41,42,43], a few characterizations of DNA modification variability among REs have been conducted, considering only 5mC modifications [44,45]. In plants, the available results indicate that REs are a preferential target of 5mC methylation compared to coding genes [46]. Interestingly, our data highlight not only that non-canonic DNA modifications are widely represented in protein coding genes as well as in the repetitive fraction (particularly in relation to LTR-REs) but also an apparent variability of 4mC and 6mA modifications between LTR-RE superfamilies, and among different LTR-RE lineages (Figure 2).

Both the *Gypsy* and *Copia* superfamilies showed higher 4mC and 6mA modification levels along their full-length elements (Figure 3) compared to the neighboring regions. Similarly to what was observed for 6mA modifications in Arabidopsis and in *Chlamydomonas reinhardtii* [30,37], the 6mA and 4mC sites exhibited a peak in the regions internal to the LTR-RE. The overall levels of DNA modifications in the coding regions were higher than in the LTRs in both the *Copia* and *Gypsy* LTR-REs.

We also analysed the 4mC and 6mA in full-length LTR-REs in relation to the chromosomal localization of the element. The density of the LTR-REs was higher near the centromeres, as regions within 3 Mbp showed the highest amount of modified bases, with an average of about two-folds higher than the distal regions (Figure 4). This was expected, because repeats are usually located in the heterochromatin regions, such as centromeres, which usually display higher levels of methylation [14].

Differences between the LTR-RE superfamilies were observed: *Copia* elements displayed lower unconventional methylation levels and a higher number of proximal genes than *Gypsy* REs. Such a difference in methylation level resonates with the distribution patterns of the two superfamilies, as *Gypsy* elements are mainly located in the centromeres and in the pericentromeric regions, where they probably play a structural role [7,47,48,49,50,51], and thus they are more prone to being controlled by methylation. However, that unconventional methylation has the same effect on LTR-RE activity and the transcription of adjacent genes than C5-cytosine methylation remains to be ascertained.

It has been reported that the high epigenetic repression of TEs through 5’-methylation of cytosine is related to a high C5-cytosine methylation level and the expression of nearby genes in different plant species [19,45,52,53], which is detrimental for the transcription of genes lying close to those chromosomal loci [54]. We studied the number of genes potentially affected by the unconventional methylation (4mC and 6mA) of LTR-REs, i.e., genes close to methylated LTR-REs (within 50,000 bp). One may expect that the higher the RE methylation level, the more the transcription repression is extended over the adjacent regions. Hence, in order to avoid gene repression, LTR-REs surrounded by many genes are less methylated than those that are adjacent to a low number of genes. As a matter of fact, in the fig genome, the level of unconventional methylation of an element is unrelated to the number of genes close to that element. This indicates that the 4mC and 6mA frequency in LTR-REs should not be related to an overall repression of transcription in the adjacency of the element. It is also possible that DNA methylation-based TE repression is related mainly or exclusively to the methylation of the carbon-5 position of cytosine, which is the most common base involved in DNA silencing [15], or to post-transcriptional mechanisms [55]. Experiments are necessary to evaluate the functional implications of the occurrence of 4mC and 6mA in the repeatome.

## 5. Conclusions

In conclusion, this study explored the unconventional DNA modification landscape in relation to the repeatome in fig, with a focus on LTR-REs.

Among the structural analyses we conducted, we explored the abundance of the different unconventional modifications that were found to be associated with the fig transposable elements, revealing that, in general, 4mC seems to be more frequent than 6mA. Focusing on LTR-REs, which represent the most abundant order of repeats in the fig genome, we inspected the different modification patterns in relation to the structure of the full-length elements, showing the ways in which the coding and LTR regions display higher modification levels than the neighboring regions.

Relating the 4mC and 6mA methylation profiles of LTR-REs with their proximity to centromeric regions allowed us to highlight how such DNA modifications decreased with the distance from the centromere, consistently with the notion that such regions display higher methylation levels. Analysing genes lying close to LTR-REs, it was observed that the high unconventional methylation level of LTR-REs did not extend to proximal genes, unlike what has been reported for the classical 5’-methylation of cytosine [19,45,52,53].

Future follow-up studies will investigate further, also analysing 5mC and using genome-wide expression data, how the expression of coding genes is affected by neighboring REs displaying high modification levels, and whether epigenetic markers can spread from repeats to genes, as was already observed in maize and rice [45,47], depending on the LTR-RE lineage, age, and chromosomal location.

## Figures and Tables

**Table 1:** Ratio of 4mC and 6mA modification and GC content % in the different types of repeats of fig.

Order	Superfamily	Lineage	4mC/6mA	GC%
**Class I**				
LTR	*Gypsy*	*Athila*	1.73	36.00
		*Chromovirus*	1.83	35.35
		*Ogre-Tat*	2.03	33.17
	*Copia*	*Ale*	1.61	39.12
		*Angela*	1.47	34.76
		*Bianca*	1.69	37.22
		*Ikeros*	1.61	32.59
		*Ivana*	1.78	35.24
		*SIRE*	1.84	36.31
		*TAR*	1.87	32.70
		*Tork*	1.27	36.12
	ND		1.71	35.93
LINE			1.63	33.88
SINE			0.96	36.79
**Class II**				
Sub-classI	*TIR*		1.74	34.99
Sub-classII	*Helitron*		1.14	37.54

## Supplementary Materials

The following are available online at https://www.mdpi.com/2223-7747/10/3/451/s1, Table S1: enriched_4mC_and_6mA_motifs_in_repeats, Table S2: genes_close_to_methylated_LTR_REs.

## Abbreviations

TE	transposon
RE	retrotransposon
LTR	long terminal repeat
5mC	5-methyl cytosine
SMRT	single-molecule real-time
PacBio	Pacific Biosciences
4mC	N4-methylcytosine
6mA	N6-methyladenine
GO	Gene Ontology

## References

[1] Wicker T., Sabot F., Hua-Van A., Bennetzen J.L., Capy P., Chalhoub B., Flavell A., Leroy P., Morgante M., Panaud O. (2007). A unified classification system for eukaryotic transposable elements. Nat. Rev. Genet..

[2] Lisch D. (2013). How important are transposons for plant evolution?. Nat. Rev. Genet..

[3] Vitte C., Fustier M.A., Alix K., Tenaillon M.I. (2014). The bright side of transposons in crop evolution. Brief. Funct. Genom..

[4] Wicker T., Keller B. (2007). Genome-wide comparative analysis of copia retrotransposons in Triticeae, rice, and Arabidopsis reveals conserved ancient evolutionary lineages and distinct dynamics of individual copia families. Genome Res..

[5] Llorens C., Futami R., Covelli L., Domínguez-Escribá L., Viu J.M., Tamarit D., Aguilar-Rodríguez J., Vicente-Ripolles M., Fuster G., Bernet G.P. (2011). The Gypsy Database (GyDB) of mobile genetic elements: Release 2.0. Nucleic Acids Res..

[6] Buti M., Moretto M., Barghini E., Mascagni F., Natali L., Brilli M., Lomsadze A., Sonego P., Giongo L., Alonge M. (2018). The genome sequence and transcriptome of *Potentilla Micrantha* and their comparison to *Fragaria Vesca* (the Woodland Strawberry). Gigascience.

[7] Neumann P., Novák P., Hoštáková N., MacAs J. (2019). Systematic survey of plant LTR-retrotransposons elucidates phylogenetic relationships of their polyprotein domains and provides a reference for element classification. Mob. DNA.

[8] Vangelisti A., Mascagni F., Giordani T., Sbrana C., Turrini A., Cavallini A., Giovannetti M., Natali L. (2019). Arbuscular mycorrhizal fungi induce the expression of specific retrotransposons in roots of sunflower (*Helianthus annuus* L.). PLoS ONE.

[9] McClintock B. (1950). The origin and behavior of mutable loci in maize. Proc. Natl. Acad. Sci. USA.

[10] Shang Y., Yang F., Schulman A.H., Zhu J., Jia Y., Wang J., Zhang X.Q., Jia Q., Hua W., Yang J. (2017). Gene deletion in barley mediated by LTR-retrotransposon BARE. Sci. Rep..

[11] Thind A.K., Wicker T., Müller T., Ackermann P.M., Steuernagel B., Wulff B.B., Spannagl M., Twardziok S.O., Felder M., Lux T. (2018). Chromosome-scale comparative sequence analysis unravels molecular mechanisms of genome dynamics between two wheat cultivars. Genome Biol..

[12] Bird A. (2007). Perceptions of epigenetics. Nature.

[13] Law J.A., Jacobsen S.E. (2010). Establishing, maintaining and modifying DNA methylation patterns in plants and animals. Nat. Rev. Genet..

[14] Reinders J., Wulff B.B.H., Mirouze M., Marí-Ordóñez A., Dapp M., Rozhon W., Bucher E., Theiler G., Paszkowski J. (2009). Compromised stability of DNA methylation and transposon immobilization in mosaic Arabidopsis epigenomes. Genes Dev..

[15] Feng S., Cokus S.J., Zhang X., Chen P.Y., Bostick M., Goll M.G., Hetzel J., Jain J., Strauss S.H., Halpern M.E. (2010). Conservation and divergence of methylation patterning in plants and animals. Proc. Natl. Acad. Sci. USA.

[16] Niederhuth C.E., Bewick A.J., Ji L., Alabady M.S., Do Kim K., Li Q., Rohr N.A., Rambani A., Burke J.M., Udall J.A. (2016). Widespread natural variation of DNA methylation within angiosperms. Genome Biol..

[17] Ong-Abdullah M., Ordway J.M., Jiang N., Ooi S.E., Kok S.Y., Sarpan N., Azimi N., Hashim A.T., Ishak Z., Rosli S.K. (2015). Loss of *Karma* transposon methylation underlies the mantled somaclonal variant of oil palm. Nature.

[18] Hollister J.D., Gaut B.S. (2009). Epigenetic silencing of transposable elements: A trade-off between reduced transposition and deleterious effects on neighboring gene expression. Genome Res..

[19] Eichten S.R., Ellis N.A., Makarevitch I., Yeh C.T., Gent J.I., Guo L., McGinnis K.M., Zhang X., Schnable P.S., Vaughn M.W. (2012). Spreading of heterochromatin is limited to specific families of maize retrotransposons. PLoS Genet..

[20] Quadrana L., Silveira A.B., Mayhew G.F., LeBlanc C., Martienssen R.A., Jeddeloh J.A., Colot V. (2016). The *Arabidopsis thaliana* mobilome and its impact at the species level. eLife.

[21] Choi K., Zhao X., Tock A.J., Lambing C., Underwood C.J., Hardcastle T.J., Serra H., Kim J., Cho H.S., Kim J. (2018). Nucleosomes and DNA methylation shape meiotic DSB frequency in *Arabidopsis thaliana* transposons and gene regulatory regions. Genome Res..

[22] Weigel D., Colot V. (2012). Epialleles in plant evolution. Genome Biol..

[23] Diez C.M., Roessler K., Gaut B.S. (2014). Epigenetics and plant genome evolution. Curr. Opin. Plant Biol..

[24] Rhoads A., Au K.F. (2015). PacBio sequencing and its applications. Genom. Proteom. Bioinform..

[25] Ye G., Zhang H., Chen B., Nie S., Liu H., Gao W., Wang H., Gao Y., Gu L. (2019). *De novo* genome assembly of the stress tolerant forest species *Casuarina equisetifolia* provides insight into secondary growth. Plant J..

[26] Usai G., Mascagni F., Giordani T., Vangelisti A., Bosi E., Zuccolo A., Ceccarelli M., King R., Hassani-Pak K., Zambrano L.S. (2020). Epigenetic patterns within the haplotype phased fig (*Ficus carica* L.) genome. Plant J..

[27] Murray I.A., Clark T.A., Morgan R.D., Boitano M., Anton B.P., Luong K., Fomenkov A., Turner S.W., Korlach J., Roberts R.J. (2012). The methylomes of six bacteria. Nucleic Acids Res..

[28] Sánchez-Romero M.A., Cota I., Casadesús J. (2015). DNA methylation in bacteria: From the methyl group to the methylome. Curr. Opin. Microbiol..

[29] Beaulaurier J., Schadt E.E., Fang G. (2019). Deciphering bacterial epigenomes using modern sequencing technologies. Nat. Rev. Genet..

[30] Liang Z., Shen L., Cui X., Bao S., Geng Y., Yu G., Liang F., Xie S., Lu T., Gu X. (2018). DNA N6-adenine methylation in *Arabidopsis thaliana*. Dev. Cell.

[31] Zhou C., Wang C., Liu H., Zhou Q., Liu Q., Guo Y., Peng T., Song J., Zhang J., Chen L. (2018). Identification and analysis of adenine N 6-methylation sites in the rice genome. Nat. Plants.

[32] Quinlan A.R., Hall I.M. (2010). BEDTools: A flexible suite of utilities for comparing genomic features. Bioinformatics.

[33] Smit A.F.A., Hubley R., Green P. RepeatMasker Open-4.0 2013–2015. http://www.repeatmasker.org.

[34] de Mendiburu F., de Mendiburu M.F. Package ‘Agricolae’. ftp://mirror.csclub.uwaterloo.ca/CRAN/web/packages/agricolae/agricolae.pdf.

[35] Studer A., Zhao Q., Ross-Ibarra J., Doebly J. (2011). Identification of a functional transposon insertion in the maize domestication gene tb1. Nat. Genet..

[36] Mori K., Shirasawa K., Nogata H., Hirata C., Tashiro K., Habu T., Kim S., Himeno S., Kuhara S., Ikegami H. (2017). Identification of *RAN1* orthologue associated with sex determination through whole genome sequencing analysis in fig (*Ficus carica* L.). Sci. Rep..

[37] Fu Y., Luo G.Z., Chen K., Deng X., Yu M., Han D., Hao Z., Liu J., Lu X., Doré L.C. (2015). N6-methyldeoxyadenosine marks active transcription start sites in *Chlamydomonas*. Cell.

[38] Zhang W., Spector T.D., Deloukas P., Bell J.T., Engelhardt B.E. (2015). Predicting genome-wide DNA methylation using methylation marks, genomic position, and DNA regulatory elements. Genome Biol..

[39] Koziol M.J., Bradshaw C.R., Allen G.E., Costa A.S., Frezza C., Gurdon J.B. (2016). Identification of methylated deoxyadenosines in vertebrates reveals diversity in DNA modifications. Nat. Struct. Mol. Biol..

[40] Liang Z., Riaz A., Chachar S., Ding Y., Du H., Gu X. (2020). Epigenetic Modifications of mRNA and DNA in Plants. Mol. Plant.

[41] Cokus S.J., Feng S., Zhang X., Chen Z., Merriman B., Haudenschild C.D., Pradhan S., Nelson S.F., Pellegrini M., Jacobsen S.E. (2008). Shotgun bisulphite sequencing of the Arabidopsis genome reveals DNA methylation patterning. Nature.

[42] Lisch D. (2009). Epigenetic regulation of transposable elements in plants. Annu. Rev. Plant Biol..

[43] Zemach A., McDaniel I.E., Silva P., Zilberman D. (2010). Genome-wide evolutionary analysis of eukaryotic DNA methylation. Science.

[44] Zakrzewski F., Schmidt M., Van Lijsebettens M., Schmidt T. (2017). DNA methylation of retrotransposons, DNA transposons and genes in sugar beet (*Beta vulgaris* L.). Plant J..

[45] Choi J.Y., Purugganan M.D. (2018). Evolutionary epigenomics of retrotransposon-mediated methylation spreading in rice. Mol. Biol. Evol..

[46] Rabinowicz P.D., Palmer L.E., May B.P., Hemann M.T., Lowe S.W., McCombie W.R., Martienssen R.A. (2003). Genes and transposons are differentially methylated in plants, but not in mammals. Genome Res..

[47] Sharma A., Presting G.G. (2008). Centromeric retrotransposon lineages predate the maize/rice divergence and differ in abundance and activity. Mol. Genet. Genom..

[48] Gong Z.Y., Wu Y.F., Koblizkova A., Torres G.A., Wang K., Iovene M., Neumann P., Zhang W., Novák P., Buell C.R. (2012). Repeatless and repeat-based centromeres in potato: Implications for centromere evolution. Plant Cell.

[49] Su H., Liu Y., Liu C., Shi Q., Huang Y., Han F. (2019). Centromere satellite repeats have undergone rapid changes in polyploid wheat subgenomes. Plant Cell.

[50] Kirov I., Odintsov S., Omarov M., Gvaramiya S., Merkulov P., Dudnikov M., Ermolaev A., Van Laere K., Soloviev A., Khrustaleva L. (2020). Functional *Allium fistulosum* centromeres comprise arrays of a long satellite repeat, insertions of retrotransposons and chloroplast DNA. Front. Plant Sci..

[51] Mascagni F., Vangelisti A., Giordani T., Cavallini A., Natali L. (2020). A computational comparative study of the repetitive DNA in the genus *Quercus* L.. Tree Genet. Genomes.

[52] Makarevitch I., Waters A.J., West P.T., Stitzer M., Hirsch C.N., Ross-Ibarra J., Springer N.M. (2015). Transposable elements contribute to activation of maize genes in response to abiotic stress. PLoS Genet..

[53] Wang G., Jiang H., de León G.D.T., Martinez G., Köhler C. (2018). Sequestration of a transposon-derived siRNA by a target mimic imprinted gene induces postzygotic reproductive isolation in Arabidopsis. Dev. Cell.

[54] Arnaud P., Goubely C., Pelissier T., Deragon J.M. (2000). SINE retroposons can be used in vivo as nucleation centers for de novo methylation. Mol. Cell. Biol..

[55] Sigman M.J., Slotkin R.K. (2016). The first rule of plant transposable element silencing: Location, location, location. Plant Cell.

