# Supplementary material for: DNA Modification Patterns within the Transposable Elements of the Fig (Ficus carica L.) Genome
Source: Plants (Basel). 2021 Feb 27;10(3):451. doi: 10.3390/plants10030451 (PMC7997441; doi:10.3390/plants10030451)
Supplement: Supplementary file 1 [file plants-10-00451-s001.zip › supplementary material Usai et al/enriched_4mC_and_6mA_motifs_in_repeats.doc]

**Supplementary information**

**Table S1.** Percentage of the most significantly enriched 4mC and 6mA motifs in the different repeat class, superfamily and lineage

| Order | Superfamily | Lineage | CG% | CHG% | CHH% | ANHGA% | GAGG% | ACCT% | CAAG% | A NO MOTIF% |
| --- | --- | --- | --- | --- | --- | --- | --- | --- | --- | --- |
| **Class I** | | | | | | | | | | |
| **LTR** | *Gypsy* | ***Athila*** | *0.32* | *0.24* | *0.40* | *0.11* | *0.04* | *0.01* | *0.04* | *0.35* |
|  |  | ***Chromovirus*** | *0.38* | *0.16* | *0.40* | *0.11* | *0.05* | *0.01* | *0.04* | *0.35* |
|  |  | ***Ogre-Tat*** | *0.67* | *0.33* | *0.54* | *0.21* | *0.06* | *0.01* | *0.03* | *0.45* |
|  | *Copia* | ***Ale*** | *0.27* | *0.15* | *0.23* | *0.07* | *0.03* | *0.01* | *0.02* | *0.27* |
|  |  | ***Angela*** | *0.39* | *0.19* | *0.37* | *0.12* | *0.04* | *0.01* | *0.03* | *0.44* |
|  |  | ***Bianca*** | *0.11* | *0.04* | *0.11* | *0.03* | *0.01* | *0.00* | *0.01* | *0.10* |
|  |  | ***Ikeros*** | *0.25* | *0.15* | *0.32* | *0.08* | *0.03* | *0.01* | *0.02* | *0.31* |
|  |  | ***Ivana*** | *0.35* | *0.13* | *0.33* | *0.09* | *0.04* | *0.00* | *0.02* | *0.30* |
|  |  | ***SIRE*** | *0.48* | *0.17* | *0.41* | *0.13* | *0.03* | *0.00* | *0.03* | *0.38* |
|  |  | ***TAR*** | *0.28* | *0.13* | *0.30* | *0.07* | *0.04* | *0.00* | *0.01* | *0.25* |
|  |  | ***Tork*** | *0.10* | *0.06* | *0.13* | *0.06* | *0.02* | *0.00* | *0.01* | *0.14* |
|  | ND |  | 0.25 | 0.12 | 0.28 | 0.08 | 0.03 | 0.01 | 0.02 | 0.24 |
| **LINE** |  |  | 0.20 | 0.08 | 0.24 | 0.07 | 0.03 | 0.00 | 0.01 | 0.20 |
| **SINE** |  |  | 0.10 | 0.06 | 0.15 | 0.08 | 0.03 | 0.00 | 0.01 | 0.21 |
| **Class II** | | | | | | | | | | |
| **Sub-classI** | ***TIR*** | | 2.18 | 1.09 | 1.90 | 0.71 | 0.22 | 0.04 | 0.10 | 1.83 |
| **Sub-classII** | ***Helitron*** | | 0.27 | 0.13 | 0.24 | 0.10 | 0.05 | 0.01 | 0.02 | 0.38 |
